# Frequency of side effects experienced in two different NIM-EMG tubes used in thyroid and parathyroid surgery; a prospective observational study

**DOI:** 10.1186/s12871-024-02643-1

**Published:** 2024-07-25

**Authors:** Oya Kale, Filiz Akaslan, Gülsen Keskin, Gökhan Toptaş

**Affiliations:** 1 Ministry of Health Ankara Etlik City Hospital, Anesthesiology and Reanimation Clinic, Varlık Mahallesi, Halil Sezai Erkut Caddesi, No: 5, Yenimahalle/Ankara, 06170 Turkey; 2 Ministry of Health Ankara Etlik City Hospital, Ear Nose Throat Clinic, Ankara, Turkey

**Keywords:** Thyroid and parathyroid surgery, NIM-EMG-ETT, Advers events

## Abstract

**Background:**

In this study, we observed the frequency of side effects encountered when the neural integrity monitor electromyogram endotracheal tube (NIM-EMG-ETT) was used in thyroidectomy and parathyroidectomy surgeries.

**Methods:**

After obtaining hospital ethics committee approval, 239 cases affiliated with the American Society of Anesthesiologists (ASA II-IV) who used NIM EMG tubes in thyroid and parathyroid surgery were included in the prospective observational study. Tube and patient-related complications encountered with two different NIM EMG-ETT (silicone and polyvinyl chloride-PVC) were recorded.

**Results:**

The average age of the patients is 49.50 ± 13.44 years, the average BMI is 28.25 ± 4.91 kg/m^2^, the median surgery time is 115 (32–475) minutes, 75.7% are women, 97.5% are ASA II. Additional diseases other than thyroid and parathyroid problems were present in 77.3%. Thyroidectomy was performed in 73.2% of the patients. In our study, only 0.8% of patients with transient recurrent laryngeal nerve RLN paralysis were observed in thyroid and parathyroid surgeries performed using NIM-EMG tubes, 3 patient already had nerve involvement in the preoperative period. The most common complication was loss of stimulation response related to tubes and patient-related ventilation failure. There was no difference between the complications of silicone and PVC tubes except for irregular EMG response.

**Conclusions:**

There was no significant difference in side effects other than irregular EMG response in the two different tubes we used in our study. It was observed that prolonging the surgical time increased the risk of irregular EMG response. It should not be forgotten that no matter which NIM-EMG tube is used, additional risks are encountered during the intubation and extubation process. In order to avoid negative consequences, it is necessary to follow the usage rules recommended by the manufacturer when using NIM-EMG tubes.

## Background

Injury to the recurrent laryngeal nerve (RLN) during thyroid and parathyroid surgeries is a potential complication. In order to cope with this complication, the electromyographic endotracheal tube (EMG-ETT) has been developed, which allows intraoperative nerve monitoring, and the “Tracheal tube, which is reinforced with 2 pairs of recording electrodes in the neural integrity monitor electromyogram (NIM-EMG)”, is positioned to contact the vocal cords, intraoperative monitoring of RLN has been used for a long time [1, 2]. Although there are positive aspects, there are also negative events reported in the literature about these tubes.

Regarding the device and the tube; cuff leakage, cuff perforation, cuff air deflation malfunction, lumen obstruction, tube defect (crack, plication or valve problem), EMG cable error, loss of stimulation response, electrode error, irregular EMG response and patient-related; Ventilation failure, airway trauma, postoperative dyspnea, allergic reaction, difficult extubation, postoperative hoarseness, bronchospasm, RLN injury, paralysis or paresis of the vocal cords, seizures, and death can be seen.

This study aimed to compare the frequency of adverse events with NIM-EMG-ETT, which has two different structures used in thyroidectomy and parathyroidectomy surgeries.

## Methods

The observational study was conducted after obtaining approval from the Health Sciences University Diskapi Yildirim Beyazit Training and Research Hospital Ethical Committee (15.08.2022/144/19). The prospective observational study was conducted at Diskapi Yildirim Beyazit Training and Research Hospital and Etlik City Hospital between August 2022 and May 2023. American Society of Anesthesiologists (ASA) II-IV 240 cases (except one case) in which NIM-EMG tubes were used during thyroid and parathyroid surgery were included in the study. Informed consent was obtained from the patients and the study was conducted in accordance with the Declaration of Helsinki.

Complications encountered with two different NIM-EMG-ETT (Medtronic reinforced standard silicone tube and Chenkang polyvinyl chloride-PVC tube) used in thyroid and parathyroid surgeries in general surgery and ENT (Ear, Nose and Throat) operating rooms were recorded in the anesthesia report. Then, the data was transferred to the study form. The patients were followed 24 h a day in the operating room, postoperative care unit and ward. Patients with loss of stimulation response were followed for 6 months.

Complications can be tube-related [cuff leakage, cuff perforation, cuff deflation malfunction, lumen obstruction, tube defect (crack, plication or valve problem), EMG cable failure, loss of stimulation response, electrode error, irregular EMG response] and patient-related (ventilation failure, airway trauma, postoperative dyspnea, allergic reaction, difficult extubation, postoperative hoarseness, bronchospasm, paralysis or paresis of the vocal cords, seizure, death).

According to the laryngoscopic image, tubes 6-6.5-7 in women and 7-7.5-8 in men were preferred by checking the cuff. The tube cuff was checked before intubation, tubes with inflation or deflation failure were replaced with a new tube. Tubes with no conduction during post-intubation control were replaced with new ones. In laryngoscopy, the electrodes were positioned in such a way that they were in contact with the vocal cords. In addition, the location of the NIM-EMG-ETT was confirmed with the device (Medtronic 3.0 EMG and Nervaana monıtor) and, if necessary, with the videolaryngoscope (C-MAC HD Monıtor and videolaryngoscope-Storz).

After attaching hemodynamic monitors to all patients, propofol, fentanyl, rocuronium were used for induction. No muscle relaxants were given after the first dose, maintenance was achieved by infusion of remifentanil and sevoflurane (1-1.5 MAC).

Neuromonitoring device was used at various stages of the operation. Electrodes were tested with translaryngeal stimulation by adjusting the monitor. Monopolar stimulation probe was used to stimulate the nerve during thyroidectomy. When the nerve was first identified, the first response to the stimulus was considered the 1st response (R1), and the response after lobectomy and bleeding control was considered the 2nd response (R2). Stimulations were first made at a level of 1–2 mA, and when no response was received, they were increased to 3 mA. Signal loss was defined as no response or low response (i.e., 100 µV or less) and absence of laryngeal twitching.

Regardless of the type of surgery, all patients who received NIM-EMG-ETT were included in the study. Multinodular goiter, completion thyroidectomies, parathyroidectomies, and thyroid tumor surgeries were included. Patients with active respiratory tract infection, foreseeable difficult airway and patients scheduled for emergency surgery were excluded from the study.

### Statistical analysis

Continuous variables were expressed as mean ± standard deviation or median (min-max), categorical data were expressed as numbers and percentages. Comparisons of categorical data were made with the Chi-Square Test or Fisher’s Exact Test. The analysis was performed with IBM SPSS version 24.0 (IBM Corporation, Armonk, NY, USA). Statistical significance level was considered as *p* < 0.05.

The number of samples was 152 when the effect size was 0.3, the alpha level was 0.05 and the power was 80%, and the number of samples was 232 when the power was 95%.

## Results

A total of 240 patients, 239 patients from the ASA II-III group and 1 patient from the ASA IV group, who had a BMI > 45 and used NIM EMG tubes for thyroid and parathyroid surgery, were included in the study. Desaturation continued after intubation with a bougie in a patient with a history of upper respiratory tract infection 1 month ago. Petechial lesions seen in the right bronchus and trachea during fiberoptic bronchoscopy were not considered to be related to the NIM-EMG tube and were not included in the study group and her surgery was postponed by waking her up. The average age of the patients is 49.50 ± 13.44 years, the average BMI is 28.25 ± 4.91 kg/m^2^, the median surgery time is 115 (32–475) minutes, 75.7% are women, 97.5% are ASA was 2 and 80.8% had mallampati score 2. Additional diseases other than thyroid and parathyroid problems were present in 77.3%. Thyroidectomy was performed in 73.2% of the patients (Table 1).

**Table 1:** Distribution of socio-demographic and clinical parameters of the patient group

	(n=239)
**Age (years) (Avg ± Ss)**	49.50 ± 13.44
**VKİ (kg/m2) (Ort ± Ss)**	28.25 ± 4.91
**Operation time (min) [median (min-max)]**	115 (32–475)
**Gender (n, %)**	
Woman Male	181 (%75,7) 58 (%24,3)
**ASA (n, %)**	
2 3 4	234 (%97,9) 4 (%1,7) 1 (%0,4)
**Mallampati (n,%)**	
1 2 3 4	36 (%15,1) 193 (%80,8) 8 (%3,3) 2 (%0,8)
**Surgery performed (n, %)**	
Thyroidectomy Parathyroidectomy Thyroidectomy+Parathyroidectomy	175 (%73,2) 42 (%17,6) 22 (%9,2)
**Comorbidities (n, %)**	
**DM (Diabetes mellitus)** **HT (Hypertension)** **Obesity (BMI of 30 > and above)**	43 (%17,9) 80 (%33,5) 62 (%25,9)

**Fig. 1:**
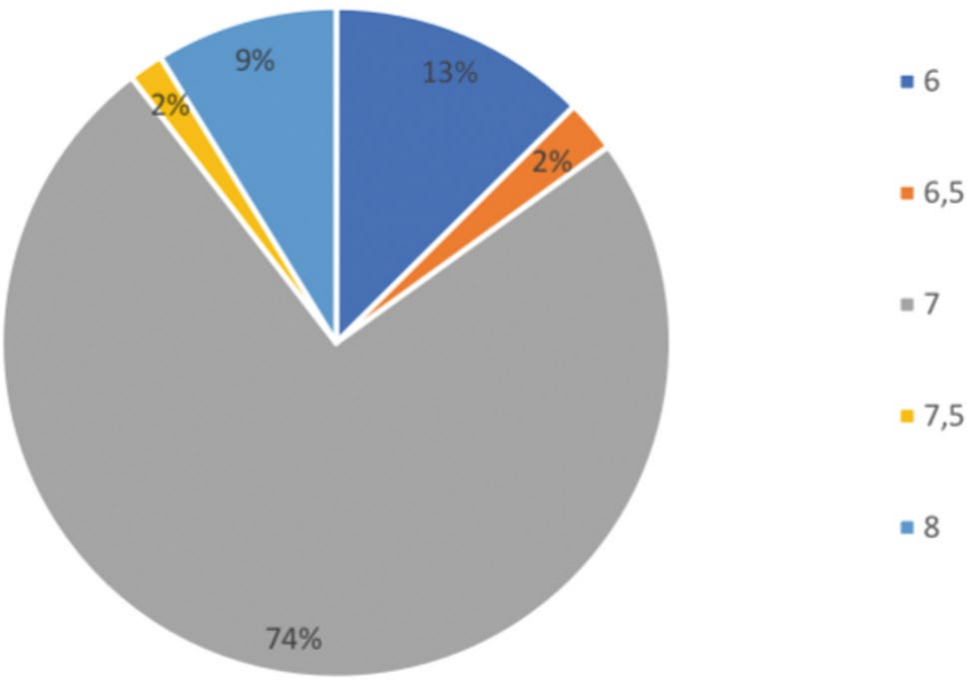
Tube numbers

Medtronic device and tube were used in 59.4% of cases, and Nerve ana device and PVC tube was used in 40.6% (Fig. 1).

**Fig. 2:**
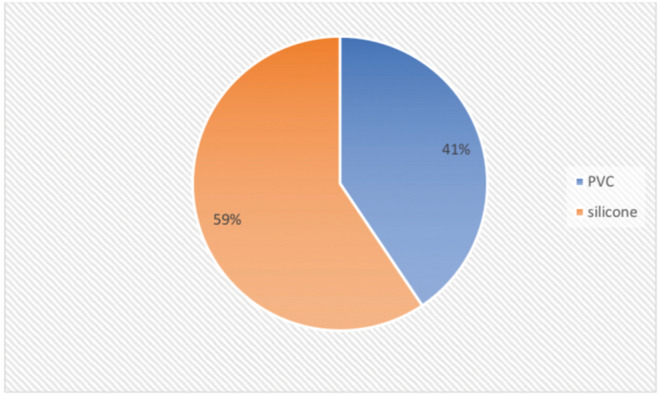
Structure of tube

It was determined that 74.5% of them used tube 7.0 (Fig. 2).

The most common complications related to the tube and the patients were loss of stimulation response (7.5%), irregular EMG response (3.3%), cuff deflation malfunction (2.1%), ventilation failure (2.1%), bronchospasm (1.3%) and transient paralysis of the vocal cords (0.8%) (Table 2). Complications such as cuff leakage, cuff perforation, tube defect, postoperative dyspnea, allergic reaction, difficult extubation, seizures, and death were not observed.

**Table 2:** Complications with the tube and the patient

	*n* (%)
**Tube related**	
Loss of stimulation response	18 (%7,5)
Irregular EMG response	8 (%3,3)
Cuff deflation malfunction	5 (%2,1)
EMG cable error	2 (%0,8)
Electrode error	1 (%0,4)
**Patient-related**	
Ventilation disorder	5 (%2,1)
Broncospasm	3 (%1,3)
Hoarseness after surgery	2 (%0,8)
Airway trauma	1(0.4%)

When the complications seen are compared according to the type of surgery;

It was determined that irregular EMG response was observed at a higher rate in patients who underwent thyroid + parathyroid (13.6%) and thyroid (2.9%) surgery than in patients who underwent only parathyroid (0.0%) surgery, and the difference was statistically significant (Table 3)

**Table 3:** Complications by type of surgery

	Group T (*n* = 175)	Group *P* (*n* = 42)	Grup T + *P* (*n* = 22)	*p*
Cuff deflation malfunction	5 (%2,9)	0 (%0,0)	0 (%0,0)	0.393*
Loss of stimulation response	15 (%8,6)	1 (%2,4)	2 (%9,1)	0.377*
EMG cable error	2 (%1,1)	0 (%0,0)	0 (%0,0)	0.692*
Electrode error	0 (%0,0)	1 (%2,4)	0 (%0,0)	0.095*
Irregular EMG response	5 (%2,9)	0 (%0,0)	3 (%13,6)	0.012*
Ventilation disorder	5 (%2,9)	0 (%0,0)	0 (%0,0)	0.393*
Airway trauma	1 (%0,6)	0 (%0,0)	0 (%0,0)	0.832*
Hoarseness after surgery	2 (%1.1)	0 (%0,0)	0 (%0,0)	0.692*
Broncospasm	3 (%1,7)	0 (%0,0)	0 (%0,0)	0.574*

* .Chi-square test.Statistically significant p-values are in bold.

T: Thyroidectomy, Parathyroidectomy, T + P: Thyroidectomy + Parathyroidectomy

When the complications encountered during the operation related to the device/tube and the patients are compared according to the type of tube used; It was determined that irregular EMG response was seen at higher rates in cases where silicone-based tubes were used, and the difference was statistically significant (Table 4).

**Table 4:** Complications according to the structure of tube used

	Silicone (n = 142)	PVC (n = 97)	*p*
Cuff deflation malfunction	1 (%0,7)	4 (%4,1)	0.161*
Loss of stimulation response	14 (%9,9)	4 (%4,1)	0.134*
EMG cable error	1 (%0,7)	1 (%1,1)	1.000*
Electrode error	1 (%0,7)	0 (%0,0)	1.000*
Irregular EMG response	8 (%5,6)	0 (%0,0)	0.023*
Ventilation disorder	5 (%3,5)	0 (%0,0)	0.082*
Airway trauma	1 (%0,7)	0 (%0,0)	1.000*
Hoarseness after surgery	1 (%0,7)	1 (%1,0)	1.000*
Broncospasm	2 (%1,4)	1 (%1,0)	1.000*

* Fisher’s Exact test. Statistically significant p-values are in bold.

There was no statistically significant difference between Mallampati score and tube number (*p* > 0.05) When complications are compared according to median surgery time; It was observed that the irregular EMG response was statistically significantly higher (*p* = 0.041) in patients with a duration of ≥ 115 min (5.7%) than in those with a duration of ≤ 115 min (0.9%). Although the loss of stimulation response was not statistically significant in patients with an operation time of ≥ 115 min, it was detected at a rate close to significance (*p* = 0.087) (Table 5). In some cases, it was observed that preoperative conduction was affected by saliva and improved with intraoral aspiration.

**Table 5:** Complications according to the duration of the operation

	Op. Duration <115 min n (115)	Op. Duration >115 min n (124)	p
Cuff deflation malfunction	2 (%1,7)	3 (%2,4)	1.000*
Loss of stimulation response	5 (%4,3)	13 (%10,6)	0.087*
EMG cable error	1 (%0,9)	1 (%0,8)	1.000*
Electrode error	1 (%0,9)	0 (%0,0)	0.483*
Irregular EMG response	1 (%0,9)	7 (%5,7)	0.041*
Ventilation disorder	1 (%0,9)	4 (%3,3)	0.371*
Airway trauma	0 (%0,0)	1 (%0,8)	0.483*
Hoarseness after surgery	1 (%0,9)	1 (%0,8)	1.000*
Broncospasm	0 (%0,0)	3 (%2,4)	0.248*

* Fisher’s Exact test. Statistically significant p-values are in bold.

## Discussion

In our study, only 0.8% of patients with transient RLN paralysis were observed in thyroid and parathyroid surgeries performed using NIM-EMG tubes, 3 patient already had nerve involvement in the preoperative period. The most common complication was loss of stimulation response related to tubes and patient-related ventilation failure. There was no difference between the complications of silicone and PVC tubes except for irregular EMG response.

RLN injury is a feared complication in neck surgery. Therefore, imaging of the RLN before proceeding with dissection is considered the gold standard. While there are publications that do not find intraoperative neuromonitoring (IONM) useful, there are also publications showing that it helps to identify the nerve and evaluate its functionality during surgery [3–6]. There are also publications showing that it reduces paralysis [7–9]. Recently RLN has become increasingly IONM has gradually entered routine use [10].

In a study of 2556 cases by Vaseliadis et al., it was shown that IONM technology significantly reduces both temporary and permanent RLN injuries, and its routine use provides surgeons with safe guidance in difficult and repetitive surgeries [11]. In a large Scandinavian database conducted by Bergenfelz et al., it was reported that there was a significant reduction in permanent RLN paralysis with the use of IONM and no bilateral paralysis [12].

According to the consensus published in 2018, it was recommended for use in cases where the risk of laryngeal nerve injury is high (anatomical variations of nerves, large goiter, bilateral thyroidectomy, thyroid cancer, and patients who have previously undergone anterior cervical neck surgery) [7]. The American Cranial Nerve Monitoring Task Force recommends its routine use in thyroidectomy according to its 2021 consensus [10].

There are different tubes for IONM, including silicone cuff and PVC. Its use requires experience, some negativities may occur. One of the adverse events reported about these tubes is ventilation failure due to cuff herniation in the perioperative period [13, 14].

The tubes used for IONM in our clinic are made of silicone cuff or PVC. The outer diameters of both tubes are larger than the same numbered standard ET. In Medtronic, the distance between the stimulation area and the cuff is longer than in standard ET tubes, and the cuff is more flexible than standard ET tubes. The stimulation area in PVC tubes is longer and the distance to the cuff is similar to standard ET tubes. Extreme care should be taken when positioning the patient who is intubated with NIM EMG tubes. During neck extension, there is a slight elongation of the trachea. In a study looking at tube depth, the optimum mean depth was found to be 20.6 +/- 0.97 cm for men and 19.6 +/- 1.0 cm for women. It was observed that taller subjects had a deeper tube depth. It is concluded that the average depth will be a useful reference for the detection and adjustment of the incorrect position of the electrodes. [15]. Carpenter et al. also encountered cuff herniation and suggested to the manufacturer that the cuff be designed to be more proximal, taking into account the length of the trachea [13]. In robotic surgery cases where these tubes are used successfully, it has been suggested that cuff balloon pressure is not a reliable indicator, that personnel should be very careful, and that the manufacturer should develop appropriate structural modifications to prevent problems. [16].

In case of any event suggestive of sudden ventilation failure in patients using NIM EMG ET, it is recommended to immediately deflate and inflate the cuff to the minimum required volume and exercise maximum caution [17].

In the case presented by Capra et al., it was observed that the flexible silicone cuff prevented ventilation with asymmetric swelling and herniation at high pressure, and bilateral pneumothorax developed after high positive pressure ventilation [18].

Difficulty in extubation has also been observed when using NIM-EMG-ET due to the inability to lower the cuff [19].

In the study by Pier et al., the most commonly reported adverse events were loss of response to nerve stimulation (34.8%), ventilation failure (25.2%), cuff perforation (18.2%), lumen obstruction (13.6%) and airway trauma (11.1%). Irregular EMG response occurred in only one patient (0.5%) and the rate of reintubation due to all events was 60.1% [20].

In our study, the most common complications in both groups were loss of response to nerve stimulation (7.5%), irregular EMG response (3.3%) and cuff deflation (2.1%). Loss of response to nerve stimulation 1. While it was 9.9% in the group, it was 4.1% in the 2nd group. There was no significant difference between the groups. Irregular EMG response was 5.6% in group 1 and 0% in group 2, which was statistically significant. Cuff deflation malfunction was more common in PVC tube, but the difference was not statistically significant. In the preoperative control, tubes with cuff discharge failure were not used. No problems were observed during extubation in the tubes used by the patients. Cuff perforation was not observed in any of the patients. Reintubation was required in 2 patients (0.8%) due to ventilation failure.

The prediction of signal loss for postoperative vocal cord paralysis is not clear. It has been reported that the signal loss in the initially dissected nerve is 90% improved in the intraoperative period [21]. Transient RLN paralysis was observed in 2 patients with signal loss, and there was no permanent paralysis. In 3 patients with a diagnosis of malignancy, unilateral RLN invasion, which was already known preoperatively, was seen both intraoperatively and confirmed by IONM device.

Although the visual integrity of RLN can be confirmed intraoperatively, many transient or permanent paralysis can be seen unexpectedly. The causes of RLN injury can be caused by transection, clamping, stretching, electrothermal injury, or ischemia and can be temporary, but it is difficult to determine the true causes of nerve injury, especially in cases where nerve integrity is not verified, where visual inspection failed during surgery.

There are also studies showing that the operation times are shortened with the use of IONM, but there is no significant change in the complication rate. It is especially recommended to be used by surgeons with little experience [22, 23]. The mean duration of operation in our study was 115 min. We observed that irregular EMG response, loss of nerve stimulation response, ventilation failure and bronchospasm were more common in surgeries exceeding this period. The prolongation of the surgical time may have been effective in increasing these complications.

In our study, ventilation failure was observed in 5 patients in whom using silicone-based tubes were used. (*p* = 0.082).

As a matter of fact, the FDA has reported that there may be a risk of airway obstruction and ventilation failure with silicone-based tubes. It is emphasized that similar reports have not been received regarding tubes made of PVC. It is requested that the company’s warnings be taken into account and that the negative events seen be reported to the manufacturer and the FDA. Prompt reporting of adverse events can help identify risks associated with medical devices [24]. Serious side effects and death due to these complications have been reported in the literatüre [20].

The company recommended that the tube cuff be checked before the surgery, that a new tube should be preferred if the cuff does not empty, and that the tubes should not be excessively bent as this may cause the electrodes to break. It has also been reported that cuff pressure and volume may change depending on the diffusion of the anesthetic gases used. It is recommended to inflate the cuff with a pressure gauge (≤ 24cmH2O) and check it intermittently intraoperatively. Another important point is that if there is manipulation after the cuff is inflated, it may cause blockage at the tube tip and/or Murphy eye with volume and displacement. It is not recommended to reuse the cuff and wires as they may deteriorate and harm the patient. The company strongly emphasized that the risk of airway obstruction can be reduced when used as directed and that the cuff should be lowered before any manipulation [25].

In the study, reintubation was performed in the 36th and 83rd minutes cases, considering that the cause of ventilation failure and bronchospasm was cuff herniation. When the removed tube was examined, it was observed that the cuff stretched and lengthened. Since patient safety was at the forefront at the time of the incident and we did not know the exact reason, we deemed it appropriate to replace the tube as soon as possible.

Surgical procedures can be hampered by a malfunction of the IONM system, which is widely accepted to improve outcomes in thyroid surgery. In order to optimize surface electrode positioning in anesthesia management, the use of videolaryngoscopes and appropriate neuromuscular block management have been suggested [26]. In some cases with difficult intubation, when we used a videolaryngoscope, the surgeon did not need to look again, as the location of the tube was visually confirmed.

## Conclusion

The use of IONM to prevent nerve damage in thyroid and parathyroid surgery is becoming widespread. Publications about NIM-EMG tubes are increasing. To avoid negative consequences, the FDA states that the manufacturer’s recommended usage guidelines for NIM-EMG tubes should be followed. There was no significant difference in side effects other than irregular EMG response in the two different tubes we used in our study. It was observed that prolonging the surgical time increased the risk of irregular EMG response. It should not be forgotten that no matter which NIM-EMG tube is used, additional risks are encountered during the intubation and extubation process.

## Data Availability

No datasets were generated or analysed during the current study.

## Abbreviations

RLN: Recurrent laryngeal nerve
ASA: American society of anesthesiologists
NIM-EMG-ETT: Neural integrity monitoring electromyogram endotracheal tube
IONM: Intraoperative neural monitoring
PVC: Polyvinyl chloride
